# A versatile interferometric technique for probing the thermophysical properties of complex fluids

**DOI:** 10.1038/s41377-022-00796-7

**Published:** 2022-04-28

**Authors:** Gopal Verma, Gyanendra Yadav, Chaudry Sajed Saraj, Longnan Li, Nenad Miljkovic, Jean Pierre Delville, Wei Li

**Affiliations:** 1 GPL Photonics Lab, State Key Laboratory of Applied Optics, Changchun Institute of Optics, Fine Mechanics and Physics, Chinese Academy of Sciences, 130033 Changchun, China; 2 School of Physical Sciences, University of Liverpool, Liverpool, L69 3BX UK; 3 Materials Research Laboratory, University of Illinois, Urbana, IL USA; 4 Department of Mechanical Science and Engineering, University of Illinois, Urbana, IL USA; 5 Department of Electrical and Computer Engineering, University of Illinois, Urbana, IL USA; 6 International Institute for Carbon Neutral Energy Research (WPI-I2CNER), Kyushu University, 744 Motooka, Nishi-ku, Fukuoka, 819-0395 Japan; 7 University of Bordeaux, CNRS, LOMA, UMR 5798, F-33405 Talence, France

**Keywords:** Optical spectroscopy, Nanophotonics and plasmonics

## Abstract

Laser-induced thermocapillary deformation of liquid surfaces has emerged as a promising tool to precisely characterize the thermophysical properties of pure fluids. However, challenges arise for nanofluid (NF) and soft bio-fluid systems where the direct interaction of the laser generates an intriguing interplay between heating, momentum, and scattering forces which can even damage soft biofluids. Here, we report a versatile, pump-probe-based, rapid, and non-contact interferometric technique that resolves interface dynamics of complex fluids with the precision of ~1 nm in thick-film and 150 pm in thin-film regimes below the thermal limit without the use of lock-in or modulated beams. We characterize the thermophysical properties of complex NF in three exclusively different types of configurations. First, when the NF is heated from the bottom through an opaque substrate, we demonstrate that our methodology permits the measurement of thermophysical properties (viscosity, surface tension, and diffusivity) of complex NF and biofluids. Second, in a top illumination configuration, we show a precise characterization of NF by quantitively isolating the competing forces, taking advantage of the different time scales of these forces. Third, we show the measurement of NF confined in a metal cavity, in which the transient thermoelastic deformation of the metal surface provides the properties of the NF as well as thermo-mechanical properties of the metal. Our results reveal how the dissipative nature of the heatwave allows us to investigate thick-film dynamics in the thin-film regime, thereby suggesting a general approach for precision measurements of complex NFs, biofluids, and optofluidic devices.

## Introduction

Nanofluids have emerged as promising heat transport fluids with enhanced thermal conductivity in a wide range of technological applications. Nanofluids are formed by suspending metallic or nonmetallic nanoparticles in a base fluid^1–4^. Nanofluids have opened a new dimension in the enhancement of heat transfer technology merely by adding <1% nanoparticle mass or volume fraction to the base fluids^1–4^. Since the first study published in 1995^1^, NFs have attracted different applications such as coolants in automobile transmission systems, electronic cooling applications, solar water heating devices, nuclear reactors, radiators, low-cost spectrally selective optical filters, etc.^4–14^. In these applications, the thermophysical properties of the nanofluid, including its heat transfer characteristics (thermal conductivity, heat capacity) and hydrodynamic properties (surface tension, viscosity), play critical roles in performance^5,15^. Therefore, the precise characterization of surface and bulk thermophysical properties of a nanofluid is indispensable in ensuring enhanced performance and predictive capability.

Various techniques have been used to explore the thermophysical properties of NFs made from nanoparticles having different shapes, sizes, the mass/volume fractions both form a theoretical and experimental approach. These include transient hot wire, temperature oscillation, and ω methods^4–19^. Among all developed methods, laser-driven thermocapillary deformation is the most promising approach to characterize the thermophysical properties of liquids due to its noninvasive, fast, and sensitive features. Thermocapillary deformation induced from localized laser heating and its delayed thermal response have been used to measure the thermal diffusivity and monitor the organic impurities in water^20–25^. However, due to its direct laser-fluid interaction, thermocapillary deformation has two outstanding challenges which limit its practical application. The first is the fact that it only works for pure fluids. When implemented for nanofluids and biofluids, a complex interplay of radiation, thermocapillarity, and scattering forces form which can lead to inaccurate determination of thermophysical properties. The second challenge is that thermocapillary deformation does not work for applications where the pump laser can lead to damage of the fluid. These include soft biological fluids^26^ or systems where the fluid is confined in a closed surface, such as heat pipes.

In this article, we address these two challenges by proposing a versatile optical technique based on pump-probe interferometry^27–30^ to characterize the thermophysical properties of both NFs and biological fluids. We show three exclusively different pump laser-induced heating configurations and demonstrate their wide applicability. Peculiarly, (1) for fluids that cannot be illuminated with the pump laser directly from the top such as biological fluids or NFs, we show that a bottom illumination configuration enables the characterization of the precise thermophysical properties. (2) For applications scenarios where bottom illumination is prohibited, and top illumination is required, such as the characterization of liquids on thick substrates like skin, we demonstrate the precise characterization of NFs by quantitatively isolating the competing effects of all three forces by taking advantage of their different time scales. (3) We show the direct measurement of NF thermophysical properties inside a closed cavity for applications mimicking heat pipes where the fluid cannot be exposed to light or the ambient environment. The measured results from our various configurations characterize the thermophysical quantities without introducing the competing effects of radiation pressure and scattering forces. These results are self-consistent and in good agreement with existing techniques. Our technique works for nearly all liquids and can be applied to a wide range of application scenarios for precise in-situ characterization of the thermophysical properties of complex fluids.

### Direct laser interactions with complex NF

**Fig. 1:**
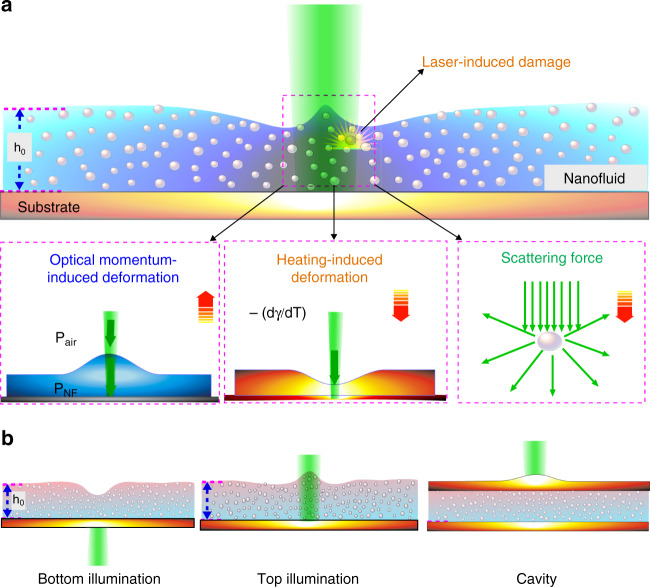
Direct laser interactions with a complex NF. **a** Schematic depicting the fundamental processes that can exist during direct laser interactions with complex NFs. The three square insets beneath the schematic illustrate the direction of interface deformation (orange arrows) induced by optical momentum (left), heating (middle), and scattering (right). Schematics not to scale. Green arrows represent the laser incident direction. The complex interplay of these processes makes it challenging to precisely characterize complex NFs using laser-based techniques. **b** Schematics of the proposed configurations including bottom illumination, top illumination, and cavity configurations

The schematic diagram shown in Fig. 1a represents the fundamental processes that are established during direct laser interaction with a complex NF. These include optical momentum-induced deformation^27,28^, scattering with nanoparticles^31,32^ in the fluid, heating-induced deformation^33–35^, as well as potential damage that may be induced by the laser, especially for soft biofluids. The surface deformation at the interface occurs as a result of these competing processes. The complex interplay of these processes makes it challenging to investigate the transient surface deformation for precise thermophysical characterization of NFs. Here, we address these challenges and demonstrate the characterization of a NF using three exclusively different types of pump laser-induced heating configurations as shown in Fig. 1b. Below we discuss each configuration and demonstrate their wide applicability.

## Results

### Pump laser illumination from the bottom of the substrate

For our first approach, we introduce a configuration where the pump laser is illuminated from the bottom of the substrate. Instead of direct laser liquid interaction, this configuration allows us to precisely characterize the thermophysical properties of complex NFs and biological fluids by eliminating scattering and radiation pressure effects. It also isolates the potential damage from the pump laser for applications when characterizing biological fluids is required^26^.

**Fig. 2:**
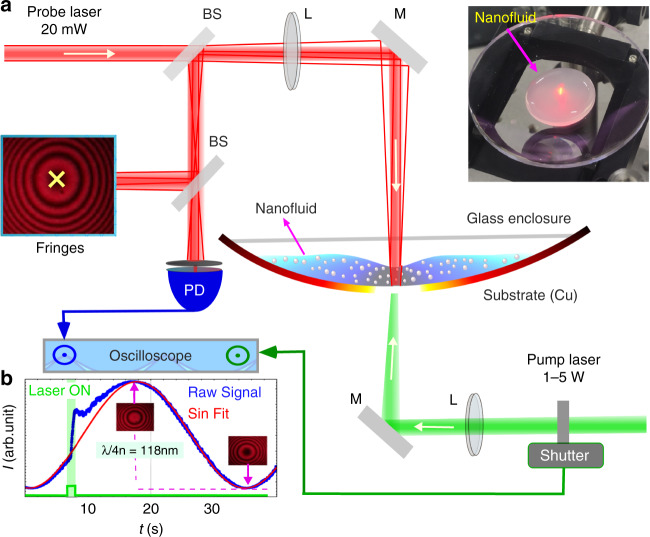
Schematic of the pump-probe setup for the bottom illumination configuration. **a** The schematic of the experimental setup. A solid-state green laser (532 nm, 1–5 W) serves as a heating beam, and a red laser (632 nm, 20 mW) probes the thermocapillary induced deformation. BS: 50:50 beam splitter, M: mirror, L: lens (focal length 200 mm), *T* = 295 K, and relative humidity 50%. Inset: Photograph of a NF during probing from the bottom. **b** An exemplary transient signal on a photodiode

In the bottom illumination setup (Fig. 2), we used a solid-state green laser. This laser serves as a heating beam for the generation of thermocapillary convection in the nanofluid by substrate heating. The power of the heating beam was varied depending on experimental requirements. The probe laser beam (He–Ne laser, *λ* = 632 nm, beam waist 1 mm) was focused on the liquid surface with a lens (focal length 200 mm) to form a Newton Ring type interference with the fringe pattern reported in refs. ^20,27–30^. The focused probe beam has a beam waist of 110 μm (see Supplementary Fig. S1). The experimental procedure includes the following steps: a water-based nanofluid made from Al_2_O_3_ or a hybrid NF (Al_2_O_3_ + Graphene-oxide (GO) nanosheet) having an initial volume fraction (*ϕ* = 0.05%) is placed on a Copper (Cu) substrate having a large radius of curvature (see “Materials and methods”). The thickness of the nanofluid layer (*h*_0_) was chosen to exclude the influence of thermo-gravitational convection in the process of thermocapillary deformation, *h*_*c*_^23^. We used a transparent glass enclosure to reduce the evaporation rate of the NF so that the concentration variation of the NF during measurement is minimized.

The evaporation of NF produced oscillations in *I*(*t*) (blue curve in Fig. 2b) which served as a reference to determine the direction (dimple/bump) of the deformation^20,27–30^. The static evaporation rate also enabled us to have different concentrations of NF for our experiment. Tracking the central intensity of the fringe *I*(*t*) allowed self-calibrating time-resolved measurement of optical thickness variation. One fringe collapse resulted in (*I*_max_ − *I*_min_) ≡ *λ*/4*n* (for *λ* = 632 nm, *n* = 1.33) = 118 nm change in the optical path length in the NF drop. By further resolving the intensity levels between the maxima and the minima, we achieved remarkable precision of ~5 nm. We have also shown the connection of the intensity signal in the center of the photodetector with the complex temporal and radial dependence of the laser-induced surface deformation in the fluid (Eqs. S1, S2), and probe beam waist dependent phase shift and in corresponding thermophysical properties change (Supplementary Figs. S2, S3).

The base fluid, nanomaterials, and human saliva initial properties are listed in Table 1, which have been obtained from refs. ^36–38^.

**Table 1:** Base fluid, nanomaterials, and human saliva properties at *T* = 295 K

Parameters	Water	Al_2_O_3_	GO	Saliva
*ρ* (kg m^−3^)	997	3987	1800	1002
*k* (W m^−1^ K^−1^)	0.6	40	3000	0.58
c_p_ (J kg^−1^ K^−1^)	4180	773	790	3760
*η* (mPa S)	0.89	—	—	1.12

When a Gaussian laser beam is incident to an absorbing Cu substrate covered with a transparent NF droplet, a nonuniform temperature distribution Δ*T*(*r, t, z*) is produced at the solid-NF interface^33–35^. The heat from this source distributes both into the depth of the substrate and the bulk of the NF layer. As a result, the isotherm will reach the free surface of the NF after a lag time^21,23^ of $\tau _d = h_0^2/4D_f$, where $D_f = k/\rho c_p$ denotes the thermal diffusivity of the fluid medium having thermal conductivity *k* and specific heat *c*_*p*_.

For small Δ*T*(*r, t, z*), all thermal properties are assumed to be constant except surface tension. The interface deformation *h* is allowed to vary linearly (Eq. S3) with the liquid temperature^20,33^. Temperature variation of the free liquid surface leads to a local decrease in surface tension, $\gamma = \gamma _0 + \gamma _T(T - T_0)$ where, *γ*_0_ is the surface tension of the liquid at a reference temperature *T*_0_, and $\gamma _T = \mathrm{d}\gamma /\mathrm{d}T$ is the surface tension coefficient, which is negative for the majority of pure liquids^21,23^. Consequently, a radially outward surface tension gradient forms along the free liquid surface, $\partial \gamma /\partial r = \gamma _T(\partial T/\partial r)\, >\, 0$ that triggers tangential thermocapillary stress along the interface directed toward the regions of highest surface tension. This force is balanced by the velocity gradient (viscous stress) in the bulk layer. This process induces a radially outward thermocapillary flow, which leads to a beam-centered dimple on top of the NF droplet interface^20^. For most fluids, including Al_2_O_3_–NF, *γ*(*T*) decreases with higher *T* (*γ*_*T*_ > 0) which leads to a thermocapillary dip^33,34^. To measure the transient height of the thermocapillary dimple *h*(*t*), we detected local interference intensity *I*(*t*) of the central fringe with a photodiode (PD) and a 2 mm aperture opening as shown in Fig. 2a.

**Fig. 3:**
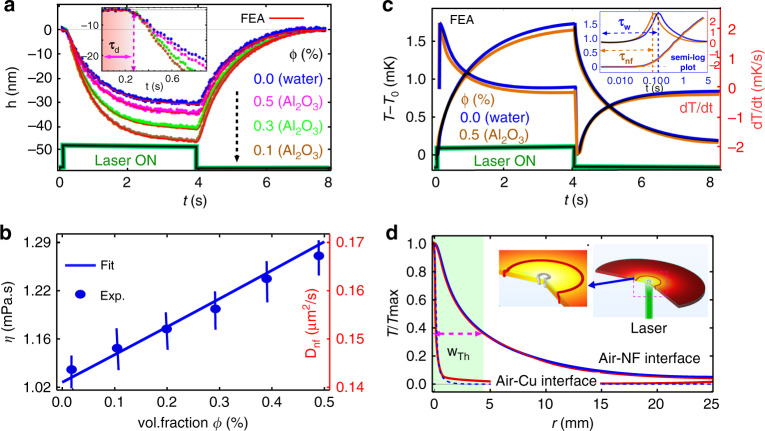
Time-resolved deformation height and numerical results for NF in a bottom illumination configuration. **a** Time-resolved surface deformation height h and shutter signal for four liquids including water (blue curve) and three Al_2_O_3_ NFs with volume fractions of 0.1% (brown curve), 0.3% (green curve), and 0.5% (pink curve). Experimental conditions: *P*_0_ = 0.2 W and w_e_ = 105 µm. The solid red lines which fit on each height curve represent finite element analysis (FEA) results. **b** Experimentally measured dynamic viscosity as a function of nanoparticle concentrations calculated from the thin-film dynamics time scale $\tau _{Th} = 3\eta w_{Th}^4/\gamma h_0^3$. **c** FEA plot of temperature variation at the fluid surface and its derivative. For clarity, only water and *φ* = 0.5% are shown. Inset: semi-log plot of the rising temperature profile. **d** Radial temperature profile at the air–NF interface. Inset: simulated surface plot of the temperature distribution

Just after switching on the pump laser beam, the probe signal did not change for a certain time interval, herein referred to as the thermocapillary delay time *τ*_*d*_ of the response. The maximum deformation *h*(*r* = 0) rapidly increases in time after the delay time *τ*_*d*_ and approaches steady state thereafter. Note that the time to reach the temperature isotherm at the Cu–NF interface for a Cu substrate thickness *h*_*Cu*_ = 300 μm is $\tau _{Cu} = h_{Cu}^2/4D_{Cu} = 9\;{\rm{ms}}$ which is very small compared to $\tau_{th}=0.82\,{\rm{ms}}$ = 160 ms. We demonstrate three different concentrations of NF particle volume fractions of *ϕ* = 0.1, 0.3, and 0.5% in water to show the experimental signal in Fig. 3a. We found that for the same experimental parameters (laser power and beam waist), the deformation height for *ϕ* = 0.1, 0.3, and 0.5% with water to have a decreasing order because *γ*(*T*) decreases with an increase in *ϕ*. The transient signal in Fig. 3a gives the *τ*_*d*_ and time constant for the thermocapillary deformation *τ*_*th*_ which are related to the diffusivity and viscosity of the NF (as shown in Fig. 3b) and water using the relation $\tau = h_0^2/(4D_f)\;{{{\mathrm{and}}}}\;{\uptau}_{{{{\mathrm{th}}}}} = 3\eta w_{th}^4/(\gamma h_0^3)$. For example, water gives $\tau _{th} = 0.82\;\mathrm{s}$. This is very large for a thick sample ($h_0 = 300\;\upmu \mathrm{m}$). We expect this such behavior as heating is a dissipative process; hence the thermal spot size increases at the liquid surface. Therefore, the dynamics of the interface deformation induced by heating starts to belong to the thin-film regime, which has a time scale^20^ of $\tau _{th} = 3\eta w_{th}^4/\gamma h_0^3$. Since we know other parameters in *τ*_*th*_ we found $w_{th} = 4.8\;\mathrm{mm}$. Hence, *h*_0_ *w*_*th*_ << 1 is satisfied.

Figure 3c shows Δ*T*(*r*, *t*, *z*) and its time derivative at the air–NF interface. The peak in the plot indicates that the present temperature gradient has become sufficiently large enough to initiate observable surface deformation at the NF–air interface. This peak also enables us to calculate the value of *τ*_*d*_. To obtain *τ*_*d*_ with higher precision, we plot the semi-log of this curve (inset of Fig. 3c), where the maxima verified our approach for this calculation. In Fig. 3d, one could easily notice that the thermal spot size at the Cu substrate and NF–air interface are different as it is broadened at the air–water interface (also at the air–NF interface, not shown) due to the diffusive nature of heat. This peculiar nature of thermal spot width variation allowed us to investigate the thick NF sample under a thin-film regime and provides reliability in our calculation of thermophysical quantities. To validate our experimental results, we performed a numerical simulation using COMSOL Multiphysics (see “Materials and methods”). We used the data from Table 1 for the base fluid, different nanoparticles, and saliva as a reference for calculating the physical properties of the NF and saliva using the mixture model^39^.

### Hybrid-nanofluids and complex biological fluids (human saliva)

To further demonstrate the wide applicability of our technique, we investigated fluids with more complex components. As a proof-of-concept demonstration, we choose a hybrid NF and a bio-fluid (human saliva) as two examples and investigated their thermophysical properties.

**Fig. 4:**
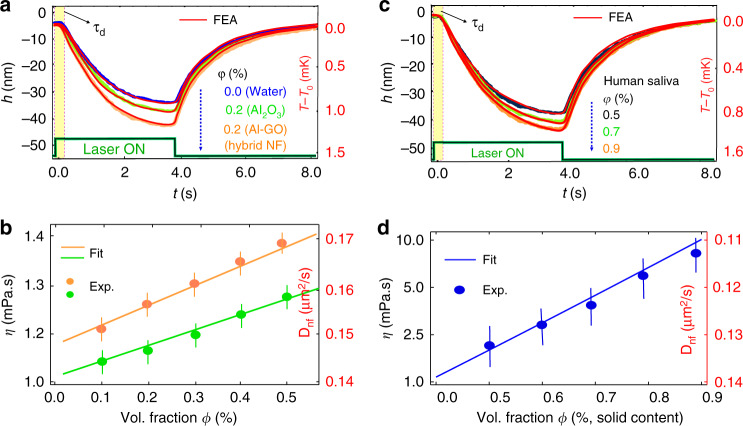
Time-resolved deformation height and numerical simulations for a hybrid NF and human saliva in the bottom illumination configuration. **a**, **c** Time-resolved deformation height and shutter signal for hybrid NF and human saliva fluids for *P*_0_ = 0.2 W and w_e_ = 105 µm. Thermal diffusivity was extracted directly by measuring the delay time (τ_d_): *D*_nf_ = *h*_*0*_^*2*^/*4τ*_*d*_. **b**, **d** Viscosity and diffusivity (right y-axis) of Al_2_O_3_ (green), hybrid NF (orange), and human saliva (blue) as a function of nanoparticle concentration. Solid lines represent lines of best fit with, $\eta = \eta _0(1 + 2.5\;\phi )$

A hybrid nanofluid is a solid-liquid mixture formed by dispersing two or more different nanoparticles suspended in a liquid, which displays enhanced thermophysical properties and rheological characteristics compared to nanofluids made from a single nanoparticle component^40–42^. We prepared an aqueous Al_2_O_3_ and graphene-oxide (GO) nanosheet hybrid NF for the characterization of thermal properties. Figure 4a presents the time-resolved deformation height and numerical results for the hybrid NF and human saliva samples. Figure 4b shows the influence of the NP volume fraction on the dynamic viscosity and thermal diffusivity of the hybrid NF, which is extracted from Fig. 4a. The results indicate that both properties of the hybrid NF increase with increased NP volume fraction in the range from 0 to 0.5%. The results show that the nanoparticle volume fraction had a significant influence on the hybrid nanofluid thermophysical properties because the thermal conductivity of GO and Al_2_O_3_ nanoparticles are larger than that of deionized water as well as the Al_2_O_3_ NF. The nanoscale sensitivity of our noninvasive optical technique to surface deformation measurement allowed us to characterize the complex biological fluid (human saliva) in a similar non-contact manner without damaging it. An absence of damage is ensured due to the micro-Kelvin scale temperature rise required to achieve a few nanometers deformation of the sample surface. Figure 4c, d shows that the transient signal of the deformed surface and extracted viscosity and diffusivity of fresh human saliva are consistent with previously reported values^38^. It is noticeable that the thermal diffusivity of saliva decreases as water content decreases. Human saliva was collected from a healthy donor, aged 25–35 years, between 9:00 and 11:00 AM. The donor refrained from eating or drinking for 2 h prior to collection. Human saliva is comprised of 99.5% water and contains many essential substances, including electrolytes, mucus, antibacterial compounds, and various enzymes. Hence for our volume concentration of saliva calculation, we considered the solid content as spherical particles of mean radius (*r*_0_).

### Top pump laser illumination on the NF surface

**Fig. 5:**
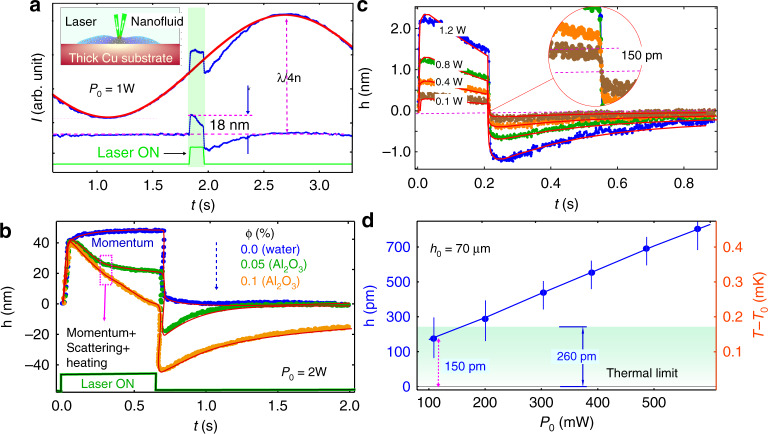
Time-resolved deformation height and numerical results for a NF with picometer sensitivity in the thin-film regime in the top illumination configuration. **a** Transient probe laser signal. Inset: schematic of the corresponding NF surface deformation induced by the pump laser. Schematic not to scale. **b** Transient surface deformation, *h(t)* measurement for water, and two different NFs having different Al_2_O_3_ NP concentrations. The solid red lines represent FEA results. **c** Time-resolved deformation height and numerical results for an NF demonstrating picometer sensitivity in the thin-film regime, for a variety of input laser powers. **d** Variation of *h(t)* and ∇*T*(*r,z,t*) (right axis) with pump power using an NF. The left inset demonstrates the time-resolved curve signal used to obtain the surface deformation and the magnification of the curve in the right inset shows the 150 pm resolutions of the measurement

We now present our second configuration using top pump laser illumination. This configuration can be used in application scenarios where bottom illumination with the laser is not easily accessible (e.g., semi-infinite substrate, structured surfaces like cloths, skin, etc…). Unlike previous work^20–25^ where the complex interplay of radiation, thermocapillarity, and scattering forces were not considered, here we show that we can precisely characterize NFs by quantitatively isolating the competing effects of all three forces by taking advantage of their different time scales. For optically transparent fluids (water) and substrates that do not absorb light at the pump laser wavelength, thermal effects are negligible on the water surface. Light momentum discontinuity results in a radiation pressure ${\Pi}_0 = \frac{2}{c}\left( {\frac{{n - 1}}{{n + 1}}} \right)I_0$, where $I_0 = 2P_0/\pi w_e^2$. The radiation pressure produces a transient nanoscale bulge having a time scale^20,29,32^ $\tau _r = 2\eta w_e/\gamma$ and an attained stationary height, $h_m\left( r \right) = \frac{{P_0\left( {n - 1} \right)}}{{\pi c\left( {n + 1} \right)}}{\int}_0^\infty {\frac{{kJ_0\left( {kr} \right)e^{ - w_e^2k^2/8}}}{{\gamma k^2 + \rho g}}dk}$ where, *J*_0_ is the zeroth-order Bessel function^20,28^. For dilute Al_2_O_3_ NF and biofluids, the absorption coefficient (*A*_*e*_) is higher than that of water. Therefore, in this case, due to weak heating, we observed a complex interplay between three forces (Fig. 5a) comprised of thermocapillary stress, radiation pressure, and volumetric force due to light scattered *f*_*scatt*_ by the nanoparticles^31,32^. In the case of spherical particles of radius *r*_0_ such that *r*_0_ << *λ*, the order of magnitude of the scattering force density involved in our experiments is inferred from the relationship *f*^*scatt*^ ≃ *τIn*/*c*^31,32^.

The turbidity (*τ*) of the NF has been found to be of the order of 50–100 m^−1^. Notice that *τ* >> *α*_*Ab*_, confirming that the light-induced force density originates mainly from light scattering. Using *n* = 1.33 as a characteristic value for the refractive index and $I_0 = 2P_0/\pi w_e^2$ with $P_0 = 0.1- 1\,\mathrm{W}$, we obtained *f*^*scatt*^ ≃ 1 × 10^2^−2 × 10^3^ N m^−3^. This force density is exerted along the direction of propagation i.e perpendicular to the NF surface, and its competing effect is shown in Fig. 5b, c. We implemented the *f*^*scatt*^ as a volumetric force in Eq. (2) (see “Materials and methods”) and radiation pressure and heating-induced surface stress at the air–water/NF interface as a boundary condition.

Radiation pressure-induced fluid deformation has a fast time scale ($\tau _r = 2\eta w_e/\gamma$) as one can see in Fig. 5b, c. The other two forces are related to the bulk flow of liquid. Therefore, radiation pressure fluid deformation appears on a different time scale and becomes distinguishable after some time of laser exposure. Figure 5b shows this expected long-term dynamics of interface deformation. Differences in time scales are even more noticeable when the pump laser is turned off. At first, the interface relaxes rapidly. Radiation pressure induces a bulge following the pump beam with steps near the rising/falling edges, as previously observed in transparent fluids^20,28,30^. Thermocapillary and scattering force-induced dimple formation exhibited slow dynamics, which were isolated as shown in Fig. 5b. The scattering force depends on the direction of incident laser light. Hence it could induce a bulge (as the radiation pressure case) on the NF surface when we shine a laser from the bottom of the glass substrate. We also performed an experiment in a thin-film NF sample for sample height (*h*_0_) about 70 μm and *ϕ* = 0.05, which allowed us to measure picometer-scale sensitivity of the NF surface deformation. Figure 5c shows the power-dependent transient probe signal which linearly depends on laser power as shown in Fig. 5d. The precision in surface deformation measurement is restricted due to the thermal limit, which is associated with the NF’s interfacial fluctuations and given by $\sqrt {k_BT/\gamma }$, where *k*_*B*_ is the Boltzmann’s constant. The value for this system is *~h* = 260 pm. Noticeably, we have measured the deformation height of ~150 pm, which is beyond the thermal limit. This displacement is measured without any lock-in or modulation on contrary to ref. ^33^ as for thin films, viscous forces dominate and suppress other fluctuations. These different degrees of precision in thick and thin films of the sample are due to bottom frictions of the substrate and gravity–capillary waves. For thick films, bottom friction vanishes and gravity–capillary waves are not damped by viscous dissipation^20^ allowing us approximately a 1 nm precision in deformation height measurement. However, bottom substrate friction becomes dominant in the thin-film regime, and gravity–capillary waves are damped by viscous dissipation, allowing us to reach picometer-scale precision.

### Nanofluid characterization inside a metallic cavity

**Fig. 6:**
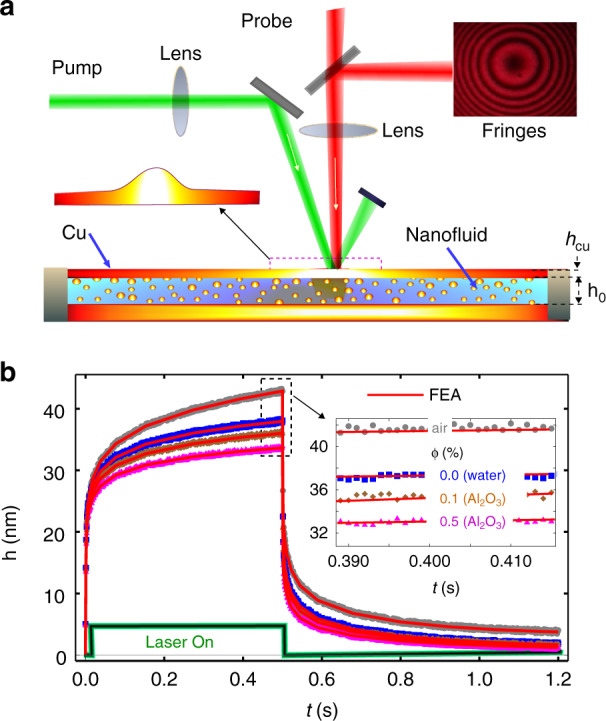
Schematic of the metal-cavity setup and measured time-resolved deformation height. **a** Schematic of the experimental setup. Schematic not to scale. **b** Measured thermoelastic deformation height *h*(*t*) of the cavity top surface (Cu–Fluid–Cu) for an air-filled cavity, water-filled cavity, and a cavity filled with two different NFs having different concentrations of NPs. Solid lines represent FEA results calculated from Eq. (3). The inset represents a reduced range view of *h*(*t*) near the saturation regime. Numerically calculated temperature and thermoelastic bulge profile of the cavity surface are also shown in Supplementary Fig. S4

We now introduce our third configuration where we show that the nanofluid inside a metallic cavity can be characterized using our approach. This configuration has its unique advantages in applications where the fluid is confined within a closed surface^35^ such as for a heat pipe, or where a fluid cannot be exposed to light or to the ambient environment. This is particularly important for applications where volatile organic compounds are present and can be absorbed by the working fluid^43–47^. It has been shown that the effect of surface heat transfer from the sample to the surroundings could be meaningful in the determination of physical parameters by laser-excited photothermal lens spectroscopy^48^. Here, we extracted the thermophysical properties of a NF by measuring the thermoelastic response of the laser-heated metal (Cu) cavity. The nanometric sensitivity of our technique allowed us to measure the laser heating-induced thermoelastic deformation (see “Materials and methods”). Figure 6a shows the schematic of the setup and surface thermoelastic response of a metal cavity filled with air, water, and two different concentrations of NFs. We observed (Fig. 6b) that the thermoelastic deformations in the cavity are higher when there is air when compared to water or NF. This is because of the increasingly higher thermal conductivity when water or NF is introduced as a fluid, compared to air. Our results could be used to obtain the physical properties of fluids having low optical absorption by using a reference solid sample in both thermal lens and thermal mirror experiments. This configuration could be useful for the noninvasive measurement of blood or any other fluid inside the viscoelastic material tube (e.g., nylon, vein/artery). Because the flow of blood or external stresses produce pressure on the vessel wall, wall deformation may occur^3^. The thermal, optical, and mechanical properties of the cavity listed below (Table 2) associated with characteristics values found from experimental data (shaded gray) and ref. ^48^ are used for the numerical simulations.

**Table 2:** Additional physical parameters obtained from the experimental data of Figs. 3 and 4

Parameters	Water	Al_2_O_3_	Al_2_O_3_ + GO	Saliva
τ_*d*_ (ms)	156	154, 148, 144	145, 143, 140	140, 155, 160
$- \frac{{{{{{d}}}}{{{\mathrm{\upsigma }}}}}}{{{{{{dT}}}}}}\;(10^{ - 3}{{{{\mathrm{N}}}}}\;{{{{{\mathrm{m}}}}}}^{ - 1}{{{{{\mathrm{K}}}}}}^{ - 1})$	0.18	0.28, 0.26, 0.23	0.16, 0.15, 0.13	0.18, 0.17, 0.16

We now discuss the self-consistency and robustness of our technique. Table 3 summarizes the measured viscosity and surface tension coefficient of a NF, hybrid NF, human saliva, and compares them with literature values. Good agreements are found for all fluids. It is also noteworthy to mention that the measured quantities under all heating configurations are consistent (see Supplementary Table S1). For example, the measured viscosity of Al_2_O_3_ NF (with φ = 0.1) in the bottom (Fig. 3a), top (Fig. 5a), and cavity (Fig. 6b) configurations are 1.14, 1.12, and 1.15 mPa s, respectively. The measured results are consistent and very close to the theoretical value of 1.13 mPa s. Furthermore, the measured quantities are independent of apparatus parameters such as substrate thickness and laser spot size, depicting the robustness of our technique.

**Table 3:** Measured dynamic viscosity and surface tension coefficient of a NF (gray shaded rows) and literature reported values for Al_2_O_3_ (ref. ^36^), Al_2_O_3_ + GO (ref. ^37^), and saliva (ref. ^38^)

Parameter	Water	Al_2_O_3_ (ϕ = 0.1, 0.3, and 0.5)	Al_2_O_3_+GO (ϕ = 0.1, 0.3, and 0.5)	Saliva (ϕ = 0.5, 0.7, and 0.9)
η (mPa s)	1.03	1.14, 1.19, 1.27	1.18, 1.25, 1.38	1.74, 3.48, 9.20
	0.95	1.13, 1.17, 1.26	1.17, 1.27, 1.39	1.75, 3.40, 9.10
$- \frac{{{{{\mathrm{d\sigma }}}}}}{{{{{\mathrm{dT}}}}}}\;(10^{ - 3}{{{\mathrm{N}}}}\;{{{\mathrm{m}}}}^{ - 1}{{{\mathrm{K}}}}^{ - 1})$	0.18	0.28, 0.26, 0.23	0.16, 0.15, 0.13	0.17, 0.15, 0.11
	0.19	0.29, 0.27, 0.21	0.17, 0.16, 0.12	0.18, 0.16, 0.13

The error bar is shown in Fig. 3

## Discussion

Four prime capabilities of our technique prove its versatility in measuring thermophysical and mechanical properties of fluids. The first capability is the nanometric sensitivity (picometer for thin-film) and self-calibrating nature of the technique. This capability allowed us to measure the time-resolved thermophysical properties of NFs, biological fluids in various configurations. The delayed thermocapillary signal directly gives the physical properties (NF and saliva) for the substrate used. An important consequence of heating the substrate from the bottom and NF inside the metal cavity is that it works for nearly all kinds of liquid as it eliminates the effects of scattering and radiation forces, as well as the potential damage induced by the laser. In addition, measuring the surface deformation beyond the thermal limit without the use of any electrical modulation distinguishes our work from past approaches. The second capability is the ability to measure weakly absorbing NFs thermal properties for a thick substrate by isolating the competing effects of thermal, scattering, and momentum transfer. The fluid dynamics can be obtained by analyzing different time scales in the surface deformation signal. This analysis can also be applicable to structured surfaces, e.g., skin, cloth, where it is difficult to shine laser from the bottom for heating^29^. The third capability is that the transient thermoelastic signal is very useful for NFs in confined environments, where heat coupling between the solid and NF provides thermophysical properties of the nanofluid only if a solid reference sample is used. The fourth capability is the noninvasive nature of the technique which is crucial for investigating the properties of complex hybrid-NFs and biological fluids^26,40^. We have demonstrated the measurement of thermophysical properties of human saliva by merely changing the temperature of the sample by a few µK and in a single measurement. Apart from this, our approach is also applicable for high concentration NFs where non-Newtonian behavior (viscoelastic) is expected^20,40,49–51^. Nanofluid films having thicknesses of a few nanometers inside the cavity would be interesting because the change in Brownian motion of the nanoparticles would play an important role in enhancing the thermal conductivity^19^.

In conclusion, our results open the possibility of applying an interferometric method for the measurement of thermophysical properties of NFs, hybrid-NFs, and biological fluids in three different types of pump laser-induced heating configurations. Future work is needed to extend the precision of our technique below 100 pm by using a twisted light beam as the probe beam^52^, that can be used to detect femto-Newton-range forces with sensitivity close to the thermal limit^53^. It would also be helpful in laser-induced cooling of solids^54^ to find the bump on NF/complex fluid surface. Our results and method have the potential to make notable contributions to opto-rheology^20^, thin-film nanometrology^55^, and designing tailored optical propulsion forces for controlled transport of resonant optical-force-guided microfluidics^56^.

## Materials and methods

### Nanofluid preparation

**Fig. 7:**
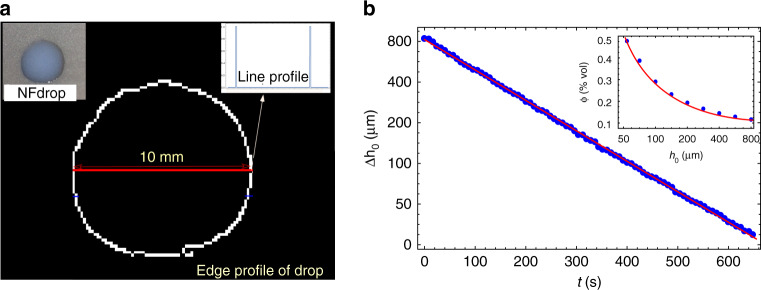
NF drop base radius and height during evaporation. **a** Drop base radius measured by analyzing the image of the droplet. Inset: photograph of the droplet and intensity profile of drop edge. **b** Measured droplet height during evaporation of the NF. Inset: NF concentration as a function of droplet height

The average diameter of Al_2_O_3_ nanoparticles (provided by Nachen, Beijing, China) is 40 nm. Morphologies of these particles are basically spherical or near-spherical. Al_2_O_3_–water nanofluids with 0.01 vol.% ~0.5 vol.% were produced by a two-step method without any surfactant. Ultrasonic vibration with an ultrasound generator (20 kHz, 100 W) was used to disperse Al_2_O_3_ nanoparticles in DI water for 3 h. Figure 7 shows photographs of Al_2_O_3_–water.

We quantified the concentration of the evaporating NF during experiments by measuring the drop height as shown in Fig. 7.

### Finite element analysis (FEA)

We performed numerical simulations with realistic sample dimensions to validate the experimental data and temperature change corresponding to deformation height. The system is symmetric around the *z* axis (incident laser beam axis). Hence, the 3D problem can be reduced to a 2D axisymmetric geometry. Two main physical mechanisms involved in the generation of thermocapillary convective flow are: heat transfer modeled by the heat equation and fluid dynamics modeled by the Navier–Stokes equation, both linked by the stress equilibrium condition $\partial \gamma /\partial r = \gamma _T(\partial T/\partial r)$. The two-phase mixture model has been extensively used for two-phase flows where the phases move at different velocities and are strongly coupled. The mixture model solves the continuity, momentum, and energy equations for the nanoparticles-fluid mixture. The momentum equation of the nanofluid is the total of the momentum equations for the fluid and nanoparticles phases. The equation of volume fraction is the continuity equation of the nanoparticle phase^39^. Thermal properties of nanofluids^39^: NF density: $\rho _{nf} = \varphi \rho _n + (1 - \varphi )\rho _f$, NF-specific heat: $\left( {\rho C_p} \right)_{nf}\, = \varphi \left( {\rho C_p} \right)_n +\, (1 - \varphi )\left( {\rho C_p} \right)_f$, NF viscosity: $\mu _{nf} = \mu _f/(1 - \varphi )^{2.5}$ and effective thermal conductivity used for input parameters for numerical simulation are defined as:

1 $$\frac{{k_{nf}}}{{k_f}} = \frac{{1 - \varphi + 2\varphi \frac{{k_n}}{{k_n - k_f}}ln\left( {\frac{{k_n + k_f}}{{2k_f}}} \right)}}{{1 - \varphi + 2\varphi \frac{{k_f}}{{k_n - k_f}}ln\left( {\frac{{k_n + k_f}}{{2k_f}}} \right)}}$$

### Temperature fields

We used the Heat Transfer in Solid Fluids module to find temperature rise distribution within the sample and substrate. This is given by the solution of the coupled heat conduction differential equation^34^, $\partial _tT_i(r,z,t) - D_i\nabla ^2T_i(r,z,t) = q_iq(r,z)$ with proper boundary and initial conditions as given in ref. ^34^. Where $D_i = k_i/\rho _ic_{pi}$ is the thermal diffusivity, *k*_*i*_ is the thermal conductivity, *ρ*_*i*_ is the mass density, and *c*_*pi*_ is the specific heat for the solid substrate (*i* = *s*) and fluid (*i* = *f*). The laser as a heat source is defined as $q_s = 2P(1 - R)\phi _{lh}/\pi c_s\rho _sw_e^2$ and $q(r,z) = \exp \left( {\frac{{ - 2r^2}}{{w_e(z)^2}}} \right)\exp \left( { - A_bz} \right)$. Since the fluid is assumed to be non-absorbing, *q*_*f*_ = 0. The portion of the absorbed power converted into heat is represented by *ϕ*_*lh*_, which in this case is ≃1. We used boundary heat source in COMSOL as a boundary condition, where *P*_0_ is the excitation power, *R* is the sample surface reflectivity. The heat flux or losses from boundaries is specified by $Q_z = - \vec n \cdot \nabla T = h_N\left( {T - T_\infty } \right) + \varepsilon \sigma _B\left( {T^4 - T_\infty ^4} \right)$ where $\vec n$ is the outward-oriented unit normal vector. *h*_*N*_ is the convective heat transfer coefficient of the substrate, *σ*_*B*_ is the Stefan–Boltzmann constant, *ε* is the emissivity of the substrate.

### Thermocapillary deformation

The Laminar Two-Phase Flow, Moving Mesh module was used to solve the Navier–Stokes equation for incompressible flow.

2 $$\rho \frac{{\partial v}}{{\partial t}} + \rho (v \cdot \nabla )v = - \nabla P + \mu \nabla ^2v + {\sum} {F_v}$$

*v* describes the flow velocity, *P* is the pressure, *ρ* is the fluid density, *μ* is the dynamic viscosity and *F*_*v*_ is the volume force. The ratio between the convective and diffusive mechanisms is given by the thermal Peclet number, which is defined as $Pe_{th} = C_pUL/k$, where *L* is a characteristic length, the height *h*_0_ of the liquid layer in this case and *U* is a characteristic velocity. The Peclet number attains a maximum of 100 at the interface near the laser spot and values below 20 elsewhere. In consequence, the convective transfer mechanism is dominant in the system, meaning that the flow velocity field will have a significant effect on the temperature field. The Maximum attained Reynolds number $Re = UL/\mu$ is obtained at the interface near the laser spot with a value of ≃10^−4^ and values below this elsewhere. This means that the convective transfer mechanism is the dominant one and the flow is considered to be laminar. We used our extracted experimental parameters as input parameters for simulation e.g., viscosity, and surface tension coefficient (see Table 1), and other thermophysical properties (density, heat capacity, and thermal conductivity) of the nanofluid using Table 4 as a function of volume fraction using the relation given in refs. ^39,50^.

**Table 4:** Base fluid, nanomaterial, and initial substrate properties at room temperature

Parameters	Units	Values
*k*_s, air, water_	(W m^−1^ K^−1^)	201, 0.026, 0.6
*D*_s, air, water_	(10^−6^ m^2^ s^−1^)	6.94, 21.9, 0.14
${{{{D}}}}_{{{{\mathrm{nf}}}}}^{\phi = 0.1,0.5}$	(10^−6^ m^2^ s^−1^)	0.14, 0.17
*ρ*_s, air, water_	(kg m^−3^)	8960, 1.17, 998
*c*_s, air, water_	(J kg^−1^ K^−1^)	384, 1005, 4180
*R*	—	0.52
*α*_T_	(10^−6^ K^−1^)	10.3
*v*_s_	—	0.31
*E*_s_	(10^9^ Pa)	120
*h*_Cu_ (top)	μm	300
*h*_Cu_ (bottom)	μm	500
*h*_0_ (fluid)	μm	1000

### Thermoelastic displacement

The Theory of Elasticity can be used to calculate the surface displacement caused by a laser-induced, nonuniform temperature distribution. In the quasi-static approximation, the thermoelastic equation^48^ is given by:

3 $$(1 - 2v)\nabla ^2u(r,z,t) + \nabla [\nabla \cdot u(r,z,t)] = 2(1 + v)\alpha _T\nabla T(r,z,t)$$

here, *u*(*r, z, t*) is the displacement vector, *v* is Poisson’s ratio, and *α*_*T*_ is the linear thermal expansion coefficient. We applied a laser heat source as a boundary condition (in Heat Transfer module) at the free surface of a Cu and “Structural Mechanics Module” to solve the above equation for thermoelastic displacement. We applied a no-slip boundary condition at the solid and fluid interface and a fixed boundary condition at the edges. As shown in Fig. 6, the experimental and FEA results are in good agreement. Initially, we experimented air-filled cavity for a given laser power 2 W and *w*_*e*_ = 105 μm in crosscheck the value of Cu properties. Then, we experimented with water and NFs.

## Supplementary information

Supporting Information

## Data Availability

The data that support the findings of this study are available from the corresponding author upon reasonable request.
