# Supplementary material for: A versatile interferometric technique for probing the thermophysical properties of complex fluids
Source: Light Sci Appl. 2022 Apr 28;11:115. doi: 10.1038/s41377-022-00796-7 (PMC9051125; doi:10.1038/s41377-022-00796-7)
Supplement: Supplementary file 1 — Supporting Information [file 41377_2022_796_MOESM1_ESM.pdf]

Supporting Information

A Versatile Interferometric Technique for Probing the Thermophysical Properties of Complex Fluids

Gopal Verma[1*], Gyanendra Yadav[2], Chaudry Sajed Saraj[1], Longnan Li[1,3], Nenad Miljkovic[3,4,5,6], Jean-Pierre Delville[7], Wei Li[1*]

[1]GPL Photonics Lab, State Key Laboratory of Applied Optics, Changchun Institute of Optics, Fine Mechanics and Physics, Chinese Academy of Sciences, Changchun 130033, China

[2]School of Physical Sciences, University of Liverpool, Liverpool L69 3BX, UK

[3]Materials Research Laboratory, University of Illinois, Urbana, IL, USA

[4]Department of Mechanical Science and Engineering, University of Illinois, Urbana, IL, USA

[5]Department of Electrical and Computer Engineering, University of Illinois, Urbana, IL, USA

[6]International Institute for Carbon Neutral Energy Research (WPI-I2CNER), Kyushu University, 744 Motooka, Nishi-ku, Fukuoka 819-0395, Japan

[7]University of Bordeaux, CNRS, LOMA, UMR 5798, F-33405 Talence, France

E-mails: gopal@ciomp.ac.cn; weili1@ciomp.ac.cn

Contents

1. Spot size of probe beam
2. Connection of the intensity signal in the center of the photodetector with the complex temporal and radial dependence of the laser-induced surface deformation in the fluid
3. Relation between surface tension coefficient and deformation height
4. Effect of probe beam size on phase shift and deformation height
5. Comparsion of measured thermophysical properties in three different configurations.
6. Temperature and thermoelastic deformation induced by the laser heating.

1. Spot size of probe beam.

We use a focusing lens (Figure 2(a) in the main text), which focus the beam about 110 μm at the drop surface.

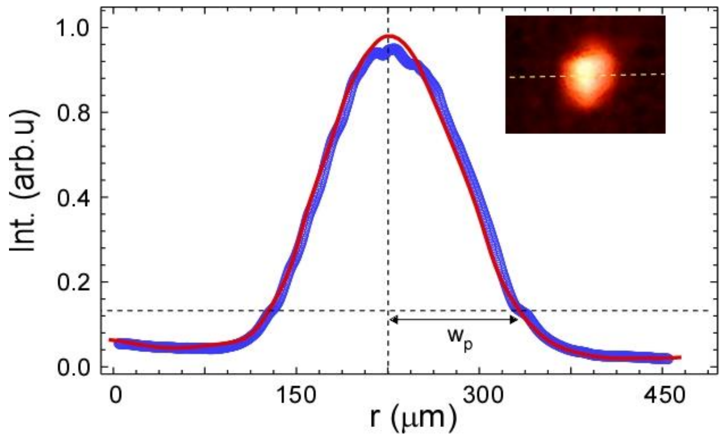

Figure. S1 Measured probe beam waist after measured after the convex lens (f = 200 mm).

This spot size (**Figure S1**) is very small compared to the curvature extension (few mm) of the bump/dip appeared at the drop. We made sure that the probe beam strike at the center of the curved surfaced. Therefore, the focusing effects on the probe reflection at the photodiode are negligible.

2. Connection of the intensity signal in the center of the photodetector with the complex temporal and radial dependence of the laser-induced surface deformation in the fluid.

The intensity of the reflected probe beam varies as:

$$I(t) = \left| \int_0^{\infty} \frac{2r}{w_p^2} \exp\left[-\left(1 + i\frac{z_1}{z_c}\right)\frac{r^2}{w_p^2} - i\Phi(r, z = 0, t) \right] dr \right|^2 \qquad (S1)$$

where z_c is the confocal distance of the probe beam, z_1 is the distance from the probe beam waist to the sample, and w_p is the radius of the probe beam at the sample surface. Neglecting the contribution of phase accumulation due to heated air Eq. S1 becomes:

$$I(t) = \left| \int_0^{\infty} \frac{2r}{w_p^2} \exp\left[-i\Phi(r, z = 0, t)\right] dr \right|^2 \qquad (S2)$$

Using the numerically calculated h (r, z=0, t) we integrate the above equation which gives the intensity at the photo diode.

3. Relation between surface tension coefficient and deformation height.

We can write simplified relation between deformation height and surface tension coefficient as reported in Ref. [33]. When a Gaussian pump beam induces a nonuniform temperature $T(r, t)$ in the liquid Figure $3(c)$, the height change of a nonuniformly heated thin-layer of liquid under gravity having temperature dependent surface-tension γ and density ρ is given by $h^2(T) = h_0^2(\rho_0/\rho)^{3/4} + 3(\gamma - \gamma_0)/\rho g$. For small variation in T, the density variation can be approximated to $\rho \simeq \rho_0$, while $\gamma(T) = \sigma_0 + \gamma_T(T - T_0)$, where γ_T is surface tension coefficient. Then height change in the thickness can be approximated as

$$h(r, T) \simeq A_0(T(r) - T_0) \qquad \text{(S3)}$$

where $A_0 = 3\gamma_T/(2\rho g h_0)$. The local depth varies linearly with $(T - T_0)$. This equation allows us to simulate the deformation profile of the drop by using numerically computed $T(r,t)$. Since we know that the sample height (h_0) and ρ and g we obtained the surface tension coefficient.

4. Effect of probe beam size on phase shift and deformation height.

Below we show the effect of probe beam's spot size on the phase shift, deformation height, and extracted thermophysical properties, considering lens/mirror effect. We numerically calculated the extension of deformation and compared it with probe beam waist as shown in Figure S2 (a,c). We have experimentally measured the effect of the probe laser beam's spot size on the phase shift and the change in deformation amplitude, for a 0.1% concentration of nanofluid, in both bottom illumination and top illumination cases (**Figure S2 (b,d)**). To do this we have measured the central intensity of fringe, keeping other parameters fixed, except probe beam waist. This gives the phase shift corresponding to path length (deformation height) change and lens/mirror effect and provide the results, considering the equivalent of interference theory. We found that in this configuration, up to 1 mm probe beam waist height causes variation in deformation about 1 nm. For top illumination (**Figure S2(c,d)**), the radial deformation extension is smaller (**Figure S2(c)**) than the bottom illumination. In this case, **Figure S2(d)** shows that up to 0.5 mm variation in spot size in this configuration causes about 1 nm change in height.

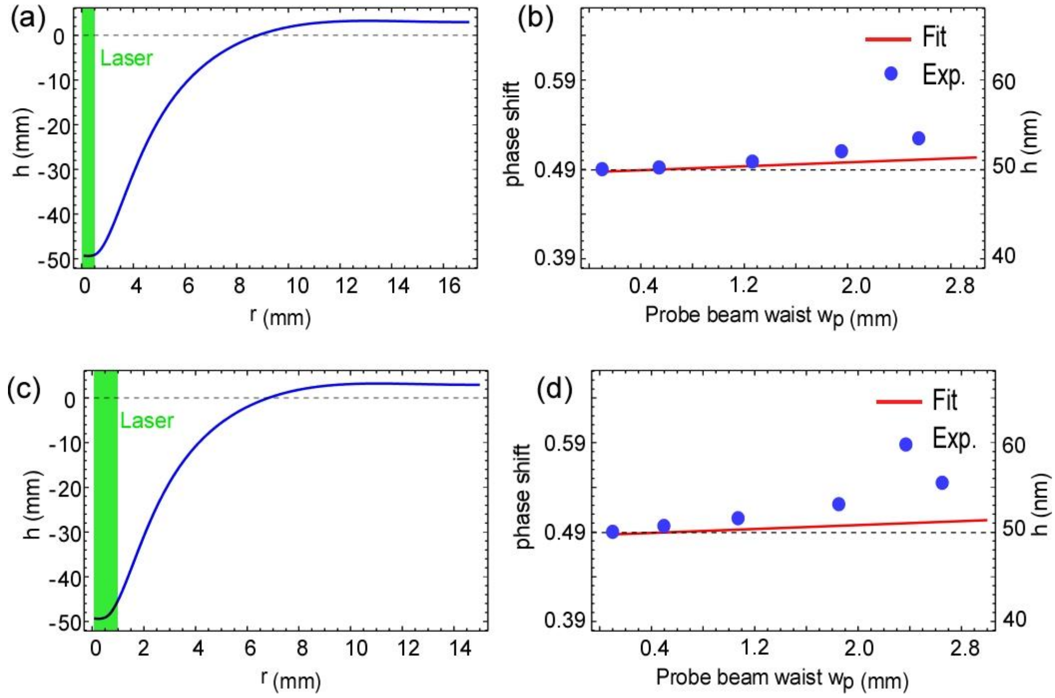

Figure S2 Extension of the deformation and effect of probe beam for a 0.1% nanofluid. (a,b). Bottom illumination (c,d) Top illumination. These plots have been chosen corresponding to the maximum deformation height on a single laser shot.

Furthermore, we have also shown that these height variation (up to 1 mm probe beam waist) has negligible effect in physical properties measurement as shown in **Figure S3**. In general, the thermal lens/mirror effect will have negligible effect on our measured results.

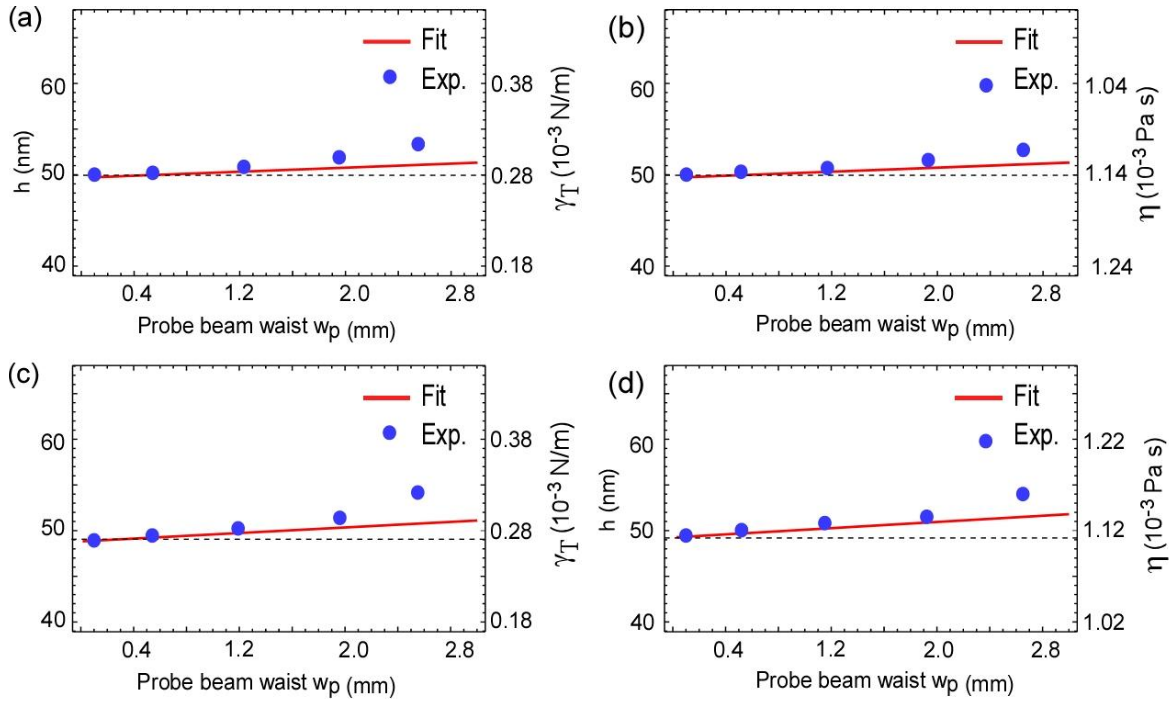

Figure S3 Deformation height and thermophysical properties (surface tension coefficient, viscosity) change as a function of probe beam waist for a 0.1% nanofluid. (a,b). Bottom illumination (c,d). Top illumination.

5. Comparison of measured thermophysical properties in three different configurations.
We have compared the measured thermophysical properties of nanofluid in three different configurations (**Table S1**). The measured results are consistent, showing the robustness of our results. It should be noted that in the cavity case there are no free liquid surfaces, hence the surface tension gradient induced thermocapillary of deformation to be zero. Therefore, the surface tension coefficients can not be measured.

**Parameters**	$\mathbf{Al_2O_3(\phi = 0.1, 0.3\ \&\ 0.5)}$		
	**Bottom**	**Top**	**Cavity**
$\boldsymbol{\eta}$ **(mPas)**	1.14,1.19,1.27	1.12,1.18,1.29	1.15,1.18,1.30
$-\frac{d\sigma}{dT}(10^{-3}N\ m^{-1}\ K^{-1})$	0.28,0.26,0.23	0.26,0.25,0.22	….

Table S1. Measured viscosity and surface tension coefficient of NF for three different concentrations.

6. Temperature and thermoelastic deformation induced by the laser heating.

We use Finite Element Analysis (FEA) based Comsol Multiphysics software for our numerical simulations. We use the Solid Fluids module to model heat transfer to find temperature distribution within the sample and substrate. The model solves the coupled heat conduction differential equation [34]. Simulation of temperature rising and thermoelastic deformation (bulge) on a metallic cavity surface are shown in **Figure S4 (a,b)**.

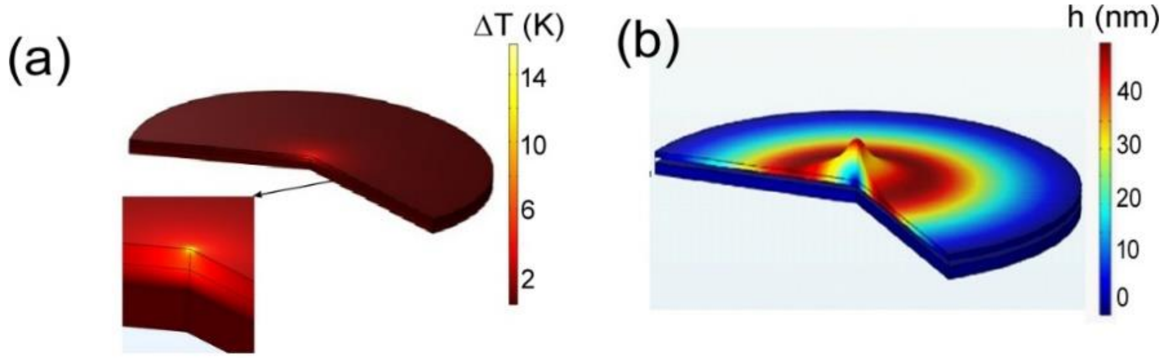

Figure S4 (a,b) Simulated temperature and thermoelastic deformation height of top Cu surface of cavity.
